# Direct Imaging of Atomic Rattling Motion in a Clathrate Compound

**DOI:** 10.1002/smsc.202300254

**Published:** 2024-02-17

**Authors:** Koudai Tabata, Takehito Seki, Scott D. Findlay, Ryo Ishikawa, Ryuji Tamura, Yuichi Ikuhara, Naoya Shibata

**Affiliations:** ^1^ Institute of Engineering Innovation School of Engineering The University of Tokyo 2‐11‐16 Yayoi Bunkyo Tokyo 113‐8656 Japan; ^2^ PRESTO Japan Science and Technology Agency 4‐1‐8 Honcho Kawaguchi Saitama 332‐0012 Japan; ^3^ School of Physics and Astronomy Monash University Wellington Road Clayton Victoria 3800 Australia; ^4^ Department of Materials Science and Technology Tokyo University of Science 6‐3‐1 Niijuku Katsushika Tokyo 125‐8585 Japan; ^5^ Nanostructures Research Laboratory Japan Fine Ceramics Center 2‐4‐1 Mutsuno Atsuta Nagoya 456‐8587 Japan; ^6^ Quantum‐Phase Electronics Center (QPEC) The University of Tokyo 2‐11‐16 Yayoi Bunkyo Tokyo 113‐8656 Japan

**Keywords:** clathrates, rattling motion, scanning transmission electron microscopy, thermal properties, thermoelectric materials

## Abstract

Controlling nanoscale heat generation, dissipation, and transport is crucial for miniaturizing electronic devices and for designing highly efficient thermoelectric materials. However, it has been challenging to directly measure thermal properties at individual atom level. Herein, direct atomic‐resolution column‐by‐column imaging of the rattling motion of Ba atoms in a clathrate compound Ba_8_Ga_16_Ge_30_ using atomic‐resolution scanning transmission electron microscopy with a segmented detector is shown. The directional anisotropy of the rattling motion is clearly visualized in real space and its amplitude and anisotropy are quantitatively evaluated by Bayesian analysis of the thermal diffuse scattering distribution. These results open a new possibility for directly characterizing nanoscale thermal properties in materials and devices, even those containing heavy elements such as thermoelectric materials.

## Graphical abstract

By multisegment detection scanning transmission electron microscopy, a quantitative observation method for atomic vibrations in heavy elements is developed. Direct atomic‐resolution imaging of the anisotropic rattling of Ba atoms within the Ba_8_Ga_16_Ge_30_ clathrate compound is delved into. Statistical image analysis precisely quantifies the amplitude and anisotropy of the atom rattling motion observed in individual atomic columns.

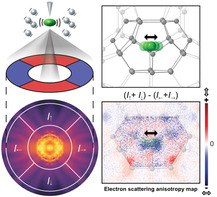

## 1 Introduction

Nanoscale thermal control is highly sought in many research fields such as thermoelectric materials^[^ ^1^, ^2^, ^3^, ^4^ ^]^, electronic devices,^[^ ^5^, ^6^ ^]^ and heat insulation materials.^[^ ^7^ ^]^ For decades, thermal control has been attempted by introducing atomic‐scale lattice defects such as impurities and interfaces because these defects have a large impact on local phonon transport properties.^[^ ^8^ ^]^ However, true understanding of thermophysical properties of atomic‐scale lattice defects is still lacking. While theoretical approaches have advanced considerably in recent years,^[^ ^9^, ^10^ ^]^ direct experimental characterization of atomic‐scale thermal properties has been challenging.

Scanning transmission electron microscopy (STEM) is a powerful technique for directly characterizing atomic‐scale lattice defects such as grain boundaries, interfaces, and point defects.^[^ ^11^ ^]^ Recent advances in monochromated electron sources have dramatically improved the energy resolution in electron energy‐loss spectroscopy (EELS), and STEM–EELS has enabled nanoscale vibrational excitation spectroscopy.^[^ ^12^, ^13^ ^]^ This provided ground‐breaking capabilities such as mapping phonon‐polariton modes,^[^ ^14^ ^]^ local measurement of phonon dispersion diagrams,^[^ ^15^, ^16^ ^]^ and detection of localized phonons at impurity atoms^[^ ^13^ ^]^ and grain boundaries.^[^ ^17^ ^]^ However, the current energy resolution of EELS still limits these measurements to the vibration of light element atoms, which have relatively large vibrational energy.

In materials such as thermoelectrics, controlling the vibration of heavy atoms is more important.^[^ ^3^, ^18^ ^]^ Clathrates are a promising compound for high‐property thermoelectric materials,^[^ ^19^ ^]^ and the thermal conductivity of Ba_8_Ga_16_Ge_30_ clathrate compounds is significantly reduced by the rattling vibration of heavy atoms (Ba) loosely bound inside a rigid cage composed of lighter atoms (Ga and Ge).^[^ ^20^, ^21^ ^]^ These heavy atoms are supposed to slowly oscillate inside the cages and scatter phonons effectively.^[^ ^22^, ^23^ ^]^ However, there is no direct experimental method to characterize local heavy atom vibrations at atomic resolution.

**Figure 1:**
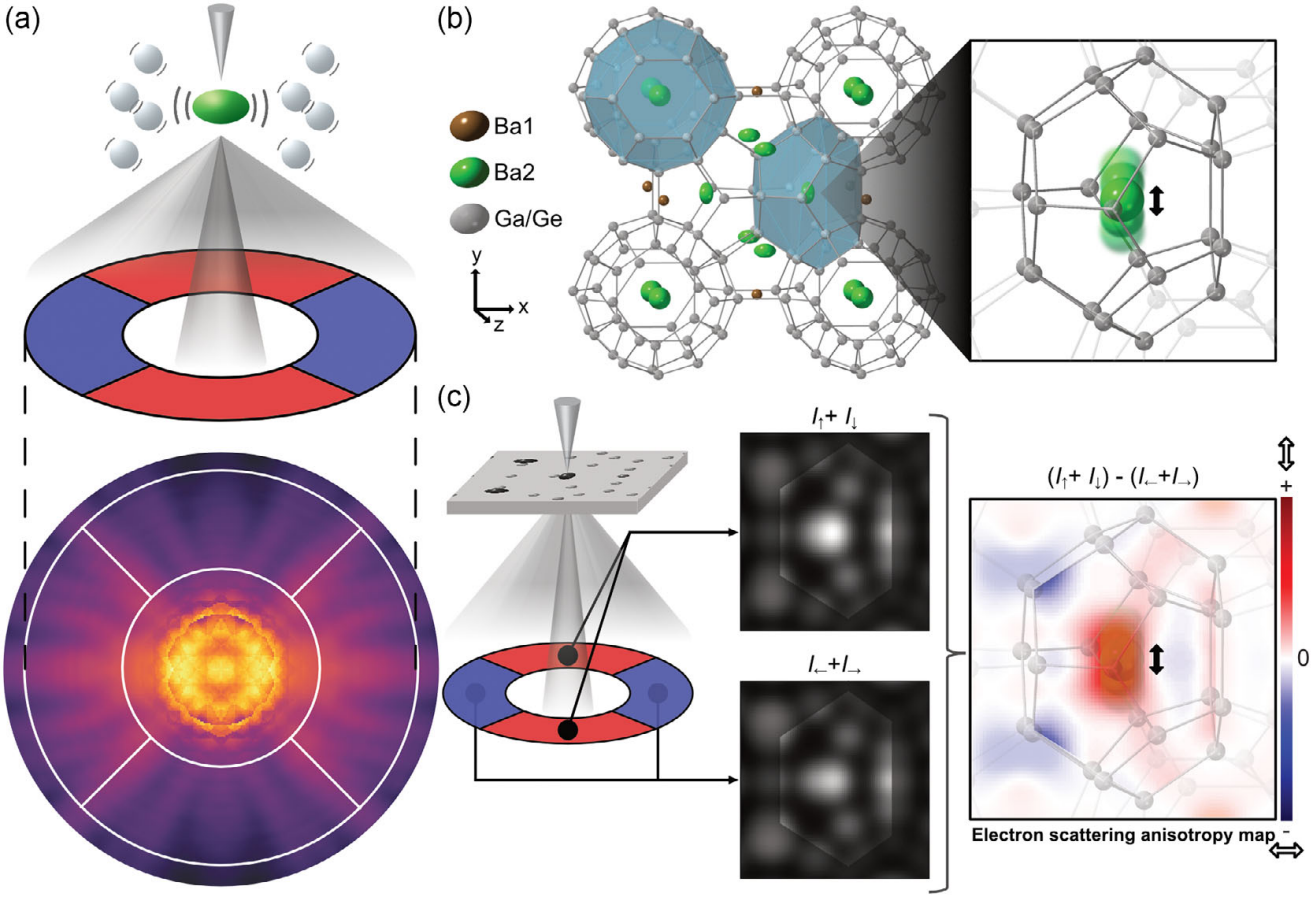
Direct imaging of anisotropic atomic vibrations by STEM. a) Schematic of using four annular segment detectors to measure anisotropy in the electron scattering intensity distribution generated by anisotropic atomic vibrations. b) Atomic structural model of Ba_8_Ga_16_Ge_30_. Within the cage (colored in blue) of the host structure, the Ba atoms at the Ba2 (6 d) rattling site, denoted in green ellipsoids, oscillate with large anisotropy. Ba1 (2a) site denoted in brown spheres is an isotropic vibrational site. c) Method of visualization of the anisotropic atomic vibrations by electron scattering anisotropy map. Large vertical electron scattering is shown in red and large horizontal scattering is shown in blue.

In STEM, direct atom imaging is commonly performed by the annular dark field (ADF) technique using a single annular detector.^[^ ^24^, ^25^, ^26^ ^]^ ADF mainly detects inelastically scattered electrons at high angles, called thermal diffuse scattering, to form atom images.^[^ ^27^, ^28^, ^29^ ^]^ Therefore, the image intensity depends on the atomic vibrational states of the atoms.^[^ ^30^, ^31^ ^]^ In recent years, a segmented detector which divides the detector plane into several segments has been developed, and multiple atomic‐resolution STEM images which are sensitive to the scattering direction and angle of electrons can be simultaneously acquired.^[^ ^32^, ^33^ ^]^ Quantitative analysis of these multiple STEM images has the potential to quantify the atomic vibrational states of individual atomic columns. In the present study, we report the real‐space column‐by‐column visualization of anisotropic atom rattling at the Ba2 sites in clathrate compound Ba_8_Ga_16_Ge_30_ (**Figure** **1**b) by atomic‐resolution dark‐field STEM with the segmented detector. Furthermore, atomic displacement parameters can be quantitatively evaluated using Bayesian estimation, which are in good agreement with other macroscopic techniques such as X‐ray and neutron scattering. This method will lead to real‐space, atomic column‐by‐column detection of local atomic vibrational anisotropy in many materials and devices.

## 2 Results and Discussion

Figure 1 shows the method of visualization of anisotropic atomic vibrations by STEM. A segmented detector was placed in the dark‐field region to detect intensity changes in the azimuthal direction caused by the anisotropic atomic vibration as schematically shown in Figure 1a. The detector was rotated so that the four segments align with the anisotropic vibrational direction of the Ba2 sites, as illustrated in Figure 1c. To visualize the anisotropy of the scattered electron distribution, the sum of the STEM images from the red‐colored detector segments was subtracted from the sum of the STEM images from the blue‐colored detector segments (see Figure 1a). We refer to the resultant difference image as an “electron scattering anisotropy map”. The site with the large vertical (y direction) oscillation, as schematically shown in Figure 1b, is expected to show strong contrast in the electron scattering anisotropy map, as schematically shown in Figure 1c.

To obtain atomic‐resolution STEM images, we used a 16‐segment photomultiplier‐tube‐based fast segmented detector, comprising four concentric annular layers each divided into quadrants (Figure S1c, Supporting Information). The accelerating voltage was 120 kV, the probe‐forming aperture was 20 mrad in semiangle, and the bright‐field disk edge was set to coincide with the outer edge of the first layer of the segmented detector (see Section 4.1 for additional details). Here, we used the third layer for imaging the anisotropy of atomic vibrations (see Figure S1d, Supporting Information). STEM images from each segment (1,024 × 1,024 pixel images, 6 μs/pixel dwell time) were acquired simultaneously in 50 consecutive scans. The images were aligned so that the positions of the same atomic columns in each image exactly coincided based on the simultaneously acquired ADF images, and then averaged to improve the signal‐to‐noise ratio. The intensity at each detector was normalized by the intensity of the incident electron beam.

**Figure 2:**
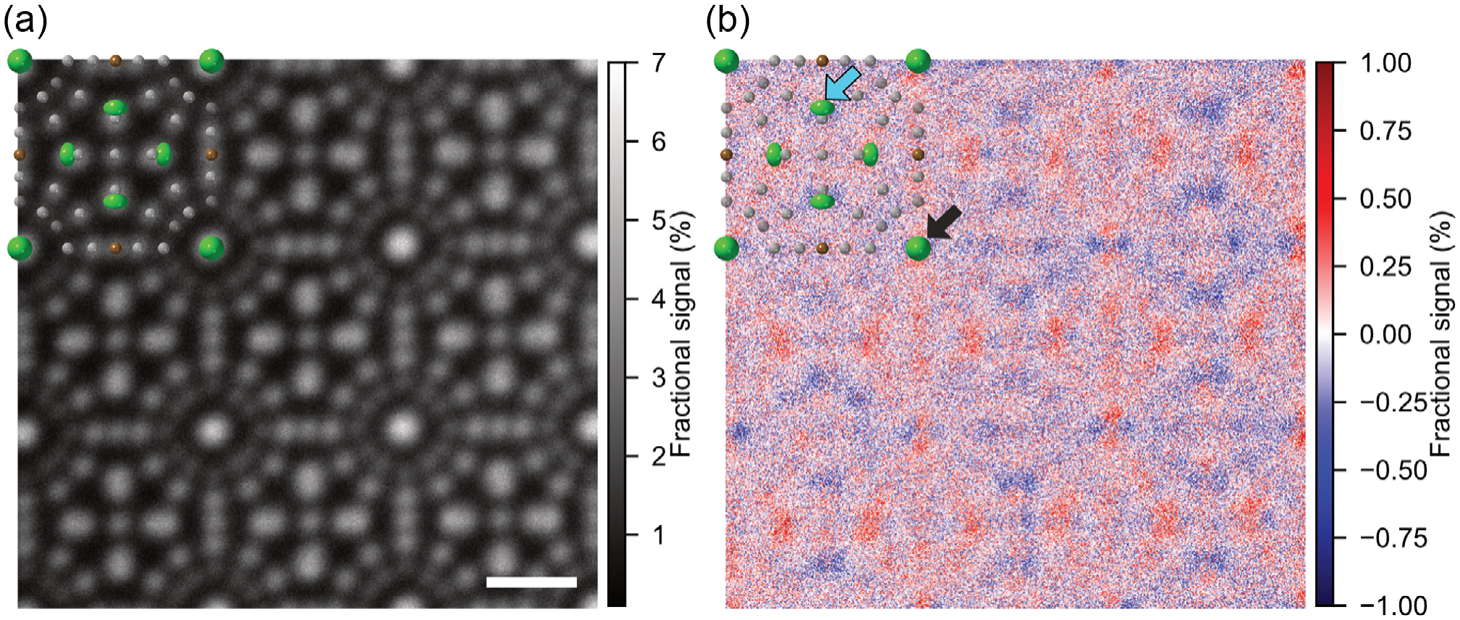
Atomic‐resolution ADF STEM image and electron scattering anisotropy map. a) ADF image and b) electron scattering anisotropy map viewed along the [001] direction. Both images were acquired in the same 40‐60 mrad scattering angle region (layer 3). The atomic structure model of Ba_8_Ga_16_Ge_30_ is overlaid on both images. Atomic shapes are shown as thermal ellipsoids to indicate atomic vibration amplitude and anisotropy. Scale bar in (a) is 5 Å. Blue and black arrows in (b) indicate Ba2 columns that have anisotropic and isotropic atomic vibrations in the in‐plane direction, respectively.

**Figure** **2**a,b shows the simultaneously acquired ADF STEM image and electron scattering anisotropy map, respectively, obtained from Ba_8_Ga_16_Ge_30_ viewed along the [001] zone axis. In the ADF image, atomic columns are visualized as bright dots, and the individual atomic column arrangements are clearly identified. The electron scattering anisotropy map shows information on electron scattering directions along the horizontal or vertical directions, at each raster point. On the Ba2 sites indicated by green ellipsoids in Figure 2b, there should be two groups of Ba atomic columns viewed along the [001] projection: one, indicated by a black arrow, with isotropic atomic vibrations within the plane of observation (the anisotropy being directed along the column), and the other, indicated by a blue arrow, with in‐plane anisotropic atomic vibrations. Cross‐shaped contrast centered on the atomic positions, with positive arms in the vertical direction and negative arms in the horizontal direction, is observed at columns with isotropic in‐plane vibration. This is due to electrons passing near an atom being scattered by the electrostatic attractive force from the nucleus in the Rutherford scattering theory.^[^ ^34^ ^]^ However, at the columns with in‐plane anisotropic atomic vibrations, the contrast indicates strong scattering anisotropy in the direction of the large atomic vibrations.

**Figure 3:**
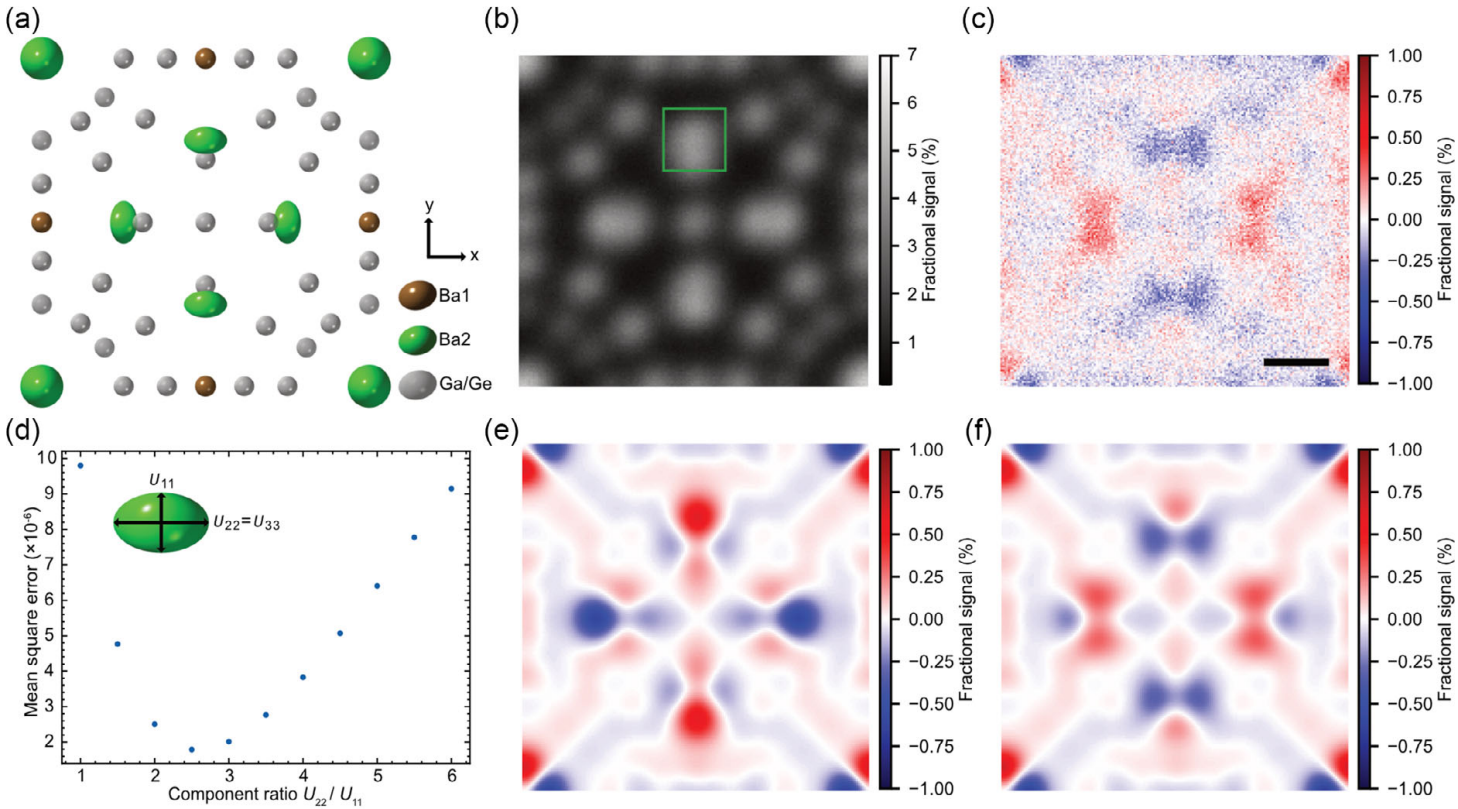
Experimental unit‐cell‐averaged electron scattering anisotropy map and simulations. a) Structural model of Ba_8_Ga_16_Ge_30_ unit cell. Experimental unit‐cell‐averaged b) ADF image and c) electron scattering anisotropy map. The green square in (b) is 2 × 2 Å^2^ centered on a Ba2 site. d) Mean square errors between the experimental and the simulated images of the electron scattering anisotropy maps for different component ratios, *U*_22_/*U*_11_, of Ba2. Simulated electron scattering anisotropy maps assuming e) isotropic and f) anisotropic atomic vibration models for the Ba2 site. In the isotropic image of (e), *U*_22_/*U*_11_ = 1. In the anisotropic image of (f), *U*_22_/*U*_11_ = 2.5. Scale bar in (c) is 2 Å.

The scattering anisotropy of single atomic columns is evident in Figure 2b. However, to improve quantitative analysis we take advantage of the periodicity evident of this structure by applying repeat‐unit averaging, based on the ADF image shown in Figure 2a, to enhance the signal‐to‐noise ratio of the STEM images. Figure S1a, Supporting Information shows repeat‐unit averaged STEM images from each detector segment and summed annular layer in 3 × 3 unit cells (3.2 × 3.2 nm). **Figure** **3**b,c shows the repeat‐unit averaged ADF image and electron scattering anisotropy map. The detailed structure in the electron scattering anisotropy map is now more clear.

However, the anisotropy of electron scattering is generally affected not only by the thermal diffuse scattering but also by the elastic scattering reflecting the crystal structure. It is therefore necessary to consider whether this contrast truly reflects the effects of atomic vibrations. We quantitatively compared the experimental electron scattering anisotropy map with STEM image simulations (see Section S2, Supporting Information, for additional details) in which the anisotropic atomic vibrations were systematically varied. The STEM image simulations were performed by varying the atomic displacement parameters of the anisotropic atomic vibration site Ba2. Ba2 site, denoted as 6 d (¼, ½, 0), exhibits a twofold degenerate within the equatorial plane (*U*_22_ = *U*_33_) and possesses a single perpendicular component, *U*_11_.^[^ ^35^ ^]^ The amplitude of the atomic displacement *U*_eq_ = (*U*_11_ + *U*_22_ + *U*_33_)*/*3 is fixed and the component ratio *U*_22_ */U*_11_ is varied to analyze the change in the electron scattering anisotropy map due to the atomic vibrational anisotropy. We computed the mean square error between the experimental and the simulated electron scattering anisotropy maps within a 2 Å square centered at Ba2, as denoted by the green box in Figure 3b. The intensities were normalized by the average intensity of the Ga/Ge columns, indicated by the blue boxes. Figure 3d shows the calculated profile of the error, with a minimum at *U*_22_ */U*_11_  = 2.5. The simulated electron scattering anisotropy map in the case of the minimum mean‐square error is shown in Figure 3f. The simulated image shows good agreement with the experimental result. For comparison, the result under the isotropic atomic vibration condition *U*_22_ */U*_11_  = 1 is shown in Figure 3e. Without the anisotropic atomic vibration, the contrast of the Ba2 site is close to the contrast of the Ba1 site, which is inconsistent with the experimental result. These results show that the electron scattering anisotropy map can detect atomic vibration anisotropy from individual atomic columns. Note that even if the atomic vibration is isotropic, the electron scattering anisotropy map may not necessarily appear to be isotropic. This is due to the contrast caused by the anisotropy of the crystal potential centered at the electron beam position.

**Figure 4:**
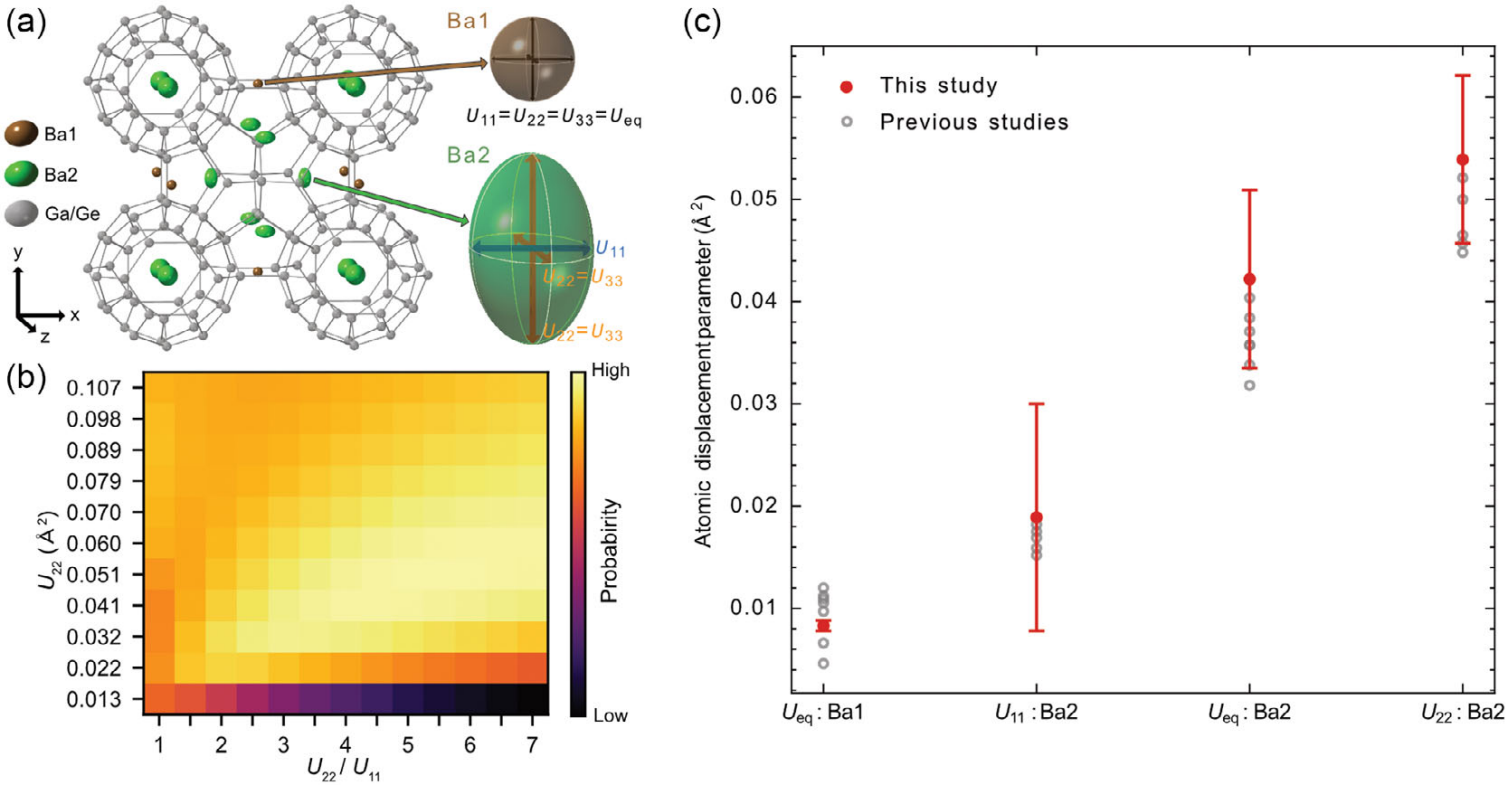
Bayesian analysis of anisotropic atomic displacement parameters. a) Atomic structural model of Ba_8_Ga_16_Ge_30_ with diagram of the direction of atomic displacement of Ba1 and Ba2 site. b) Heatmap of the posterior probability of Bayesian estimation in Ba2 site. The cross sections for *U*_22_ and *U*_22_ /*U*_11_ are shown here. c) Comparison between atomic displacement parameters of Ba atomic sites estimated in this study and the previous reports using X‐ray and neutron diffraction. Components of the atomic displacement parameter at room temperature in Ba1 and Ba2 sites are shown. The red color‐filled markers are from this study and the gray open markers are from the previous studies.^[^ ^35^, ^36^, ^37^, ^38^, ^39^ ^]^ The error bars show the standard errors.

To extract more quantitative information, we performed Bayesian estimation (see Section 4.2 for additional details) on four images: the ADF image and the electron scattering anisotropy map from the 40–60 mrad scattering angle region (layer 3) and the ADF image and the electron scattering anisotropy map from the 60–80 mrad scattering angle region (layer 4). Posterior probabilities of parameters were calculated by systematically varying three parameters: the two components of the atomic displacement parameters (the large atomic displacement *U*_22_  = *U*_33_ and the small atomic displacement *U*_11_) and the site occupancy. **Figure** **4**b shows a heat map of the posterior probability of the parameters (*U*_22_ and *U*_22_ */U*_11_) in a certain Ba2 column, with bright areas indicating high probability (see Figure S2a–c, Supporting Information for results of other cross sections for the parameters). We find an unambiguous parameter combination that maximizes the posterior probability and take these as our parameter estimates. We independently estimated these parameters for the equivalent atomic columns before the repeat‐unit averaging and calculated the mean and standard error as the uncertainty of the estimation (see Section 4.3 for additional details). We quantitatively estimate the site occupancy to be 0.669 ± 0.010, and the atomic displacement parameters to be *U*_22_ = *U*_33_ = 0.0539 ± 0.0082 Å^2^ and *U*_11_ = 0.0189 ± 0.0111 Å^2^. Similar Bayesian estimation was performed for Ba1, resulting in the occupancy of 0.763 ± 0.008 and the atomic displacement parameter of 0.0083 ± 0.0005 Å^2^ (see Section S3, Supporting Information, for additional details). Figure 4c shows that the estimated atomic displacement parameters agree very well with those from previous reports using averaging techniques such as X‐ray and neutron diffraction.^[^ ^35^, ^36^, ^37^, ^38^, ^39^ ^]^ These results indicate that the present method is capable of quantitatively estimating the atomic displacement parameters from very localized regions and from individual atomic columns in real space.

## 3 Conclusion

In summary, we have demonstrated real‐space imaging of atomic vibrations using STEM with a segmented detector. Anisotropic atom rattling motion of Ba atomic columns in a clathrate compound (Ba_8_Ga_16_Ge_30_) was visualized with atomic resolution. Bayesian parameter estimation was employed to quantitatively determine the atomic displacement parameters of individual atomic sites by quantitative comparison between the experimental and the simulated images. Here, we emphasize that the present results open a new avenue for atomic‐scale observation of thermal vibrations of heavy elements, leading to a better understanding and control of atomic‐scale phonon transport phenomena in thermoelectric materials such as clathrate materials. This method is expected to evolve into a potent technique for the visualization and quantification of thermal vibrations of heavy elements from localized regions, including surfaces and interfaces.

## 4 Experimental Section

4.1

4.1.1

##### Electron Microscopy Experiments

To remove hydrocarbon contamination,^[^ ^40^ ^]^ the TEM grid was annealed overnight at 300 °C in a high vacuum TEM in an in situ TEM heating holder (JEOL, Ltd.), and then cooled down to room temperature. STEM images were acquired with a JEM ARM300CF installed at the University of Tokyo, equipped with a JEOL DELTA corrector,^[^ ^41^ ^]^ cold field emission gun, and a second‐generation segmented annular all‐field detector (SAAF, 16 segment elements)^[^ ^32^, ^33^ ^]^ operated at 120 kV. The probe‐forming aperture was selected to be 20 mrad in semiangle and the bright‐field disk edge was set to coincide with the outer edge of the first layer of the SAAF detector (see Figure S1c,d, Supporting Information for an explanation of detector layers). Under this condition, the intensity variation due to the anisotropy of atomic vibrations becomes significant at the third detection layer (40–60 mrad). We meticulously adjusted the defocus value to maximize the intensity of the divergence, or charge density, of the differential phase contrast images in the live divergence imaging mode.

To accurately measure the probe current considering the decay of the emission current of the cold field emission gun, the beam intensity was measured immediately after the experimental image acquisition by acquiring a low‐mag image including the vacuum region. For converting the detected intensity of the bright field into beam current, we prepared references. In this method, a converged probe of a premeasured current (using a Faraday cup) scans the detector. Using this method, the probe current was estimated to be 9.8 pA. The image intensities detected with the sample present are in the linear response range, suggesting that the detector scan could be used to convert the units of intensity of each detected segment into the units of electron current. For quantitative comparison with image simulations, the background level was removed by subtracting the noise level of the vacuum and the image intensity contribution of the amorphous surface, and the units of detected intensity were finally converted into fractional intensity.

##### Bayesian Estimation

We explore atomic parameters by directly comparing intensity profiles between the experiments and the simulations based on simplified Bayesian statistical analysis.^[^ ^42^ ^]^ Let $I_{i}\in \left\{I\right\}$ be the number of electrons detected at pixel i in the STEM image I, and θ be the physical property parameter to be estimated. The posterior probability that the parameter is θ for an intensity $I_{i}$ is given by

(1) $$P\left(\theta |I_{i}\right)=\frac{P(I_{i}|\theta )}{\int P(I_{i}|\theta )P(\theta )d\theta }P(\theta )$$

As the likelihood $P\left(I_{i}|\theta \right)$ is equivalent to the output of a noise‐aware simulation with parameter θ, $P\left(I_{i}|\theta \right)$ is expressed as follows

(2) $$P\left(I_{i}|\theta \right)=\frac{1}{\sqrt{2\pi \sigma^{2}}}\exp\left(-\frac{{\left(I_{i}^{\text{ex}}-I_{i}^{\text{sim}}(\theta )\right)}^{2}}{2\sigma^{2}}\right)$$

where $I_{i}^{\text{ex}}$ is the experimental intensity at pixel i and $I_{i}^{\text{sim}}\left(\theta \right)$ is the simulated intensity with the parameter of θ. To consider shot noise, it was assumed that the distribution of intensities with respect to the simulated values follows a Gaussian distribution with constant variance, a good approximation to a Poisson distribution for the present number of counts. Assuming a uniform prior distribution P(θ) and taking the intensity to also be the variance of the Gaussian distribution (as per Poisson counting statistics), Equation (2) can be transformed as follows

(3) $$P\left(\theta |I_{i}\right)\propto P\left(I_{i}|\theta \right)\propto \exp\left(-\frac{{\left(I_{i}^{\text{ex}}-I_{i}^{\text{sim}}(\theta )\right)}^{2}}{2I_{i}^{\text{ex}}}\right)$$

When the experimental value $I_{i}^{\text{ex}}\in \left\{I^{\text{ex}}\right\}$ is obtained, the statistical noise does not correlate between the different pixels (put another way, each probe position is effectively an independent experiment). Therefore, the probability of obtaining the parameter θ can be determined as follows

(4) $$P\left(\theta |I^{\text{ex}}\right)=\prod_{i}P\left(\theta |I_{i}^{\text{ex}}\right)\propto \prod_{i}\exp\left(-\frac{{\left(I_{i}^{\text{ex}}-I_{i}^{\text{sim}}(\theta )\right)}^{2}}{2I_{i}^{\text{ex}}}\right)$$

In essence, we can assess the probability of the parameters by comparing the experimental intensities with the noise‐aware simulated intensities. This method allows the comparison of detection intensities in several detection segments in a segmented detector. This is because the signals in each segment are considered uncorrelated with each other and each probability in each detection segment follows an independent distribution.^[^ ^43^ ^]^ Therefore, by summing the variances of each segment, this method can be applied to both ADF images by summing the segments and anisotropic maps by taking the difference of the segments. By calculating the simultaneous probabilities applied to each defined image, we can account for both the anisotropy of the intensity distribution and the variation in the direction of the scattering angle during parameter estimation.

##### Uncertainty Estimation

We performed statistical uncertainty analysis of the estimation of the atomic displacement parameters based on the STEM images without repeat‐unit averaging. First, Wiener filtering was applied to the unit‐cell‐averaged ADF image, and a 0.8 Å square region for Ba1 and a 2 Å square region for Ba2 were cut out of the image as templates (see Figure S3b, Supporting Information). Using the templates, template matching by cross‐correlation function was performed on the images before averaging (Figure S3c, Supporting Information) to obtain six regions for Ba1 and nine regions for Ba2. The same Bayesian estimation as in the main text was performed on the resulting multiple atomic columns to determine the atomic displacement parameters and the site occupancy rates in each column. Uncertainty estimates were made by calculating the mean and standard error of those results.

## Conflict of Interest

The authors declare no conflict of interest.

## Supporting information

Supplementary Material

## Data Availability

The data that support the findings of this study are openly available in Zenodo at http://doi.org/10.5281/zenodo.8418310, reference number 8418310.
