# Supplementary material for: Direct Imaging of Atomic Rattling Motion in a Clathrate Compound
Source: Small Sci. 2024 Feb 17;4(4):2300254. doi: 10.1002/smsc.202300254 (PMC11935144; doi:10.1002/smsc.202300254)
Supplement: Supplementary file 1 — Supplementary Material [file SMSC-4-2300254-s001.pdf]

Supporting Information

Direct Imaging of Atomic Rattling Motion in a Clathrate Compound

Koudai Tabata, Takehito Seki, Scott D. Findlay, Ryo Ishikawa, Ryuji Tamura, Yuichi Ikuhara, Naoya Shibata**

E-mail: seki@sigma.t.u-tokyo.ac.jp, shibata@sigma.t.u-tokyo.ac.jp*

This PDF file includes:

Section S1-4

Figure S1-5

Section S1. Sample preparation

A polycrystalline alloy with a nominal composition of $Ba_8Ga_{16}Ge_{30}$ was synthesized from high-purity Ba (99.9 wt.%), Ga (99.9999 wt.%) and Ge (99.99 wt.%) elements using an arc-melting technique. The obtained sample was ground in an agate mortar and then phase identification was performed by powder X-ray diffraction using *K*a radiation (Rigaku MiniFlex600). The crushed samples were placed on Mo TEM grids with carbon layers for STEM observation.

Section S2. Image simulation method

In this study, we employed the multislice method to simulate STEM images. The microscope parameters used here are consistent with the experimental values. A defocus was set in the simulations with the same value for the experiment: 4 nm underfocus. The detector angles and orientation relative to the $Ba_8Ga_{16}Ge_{30}$ single crystal were consistent with the experimental setup. Based on the low-angle ADF images (Figure S4), the sample thickness was estimated to be 8 nm. Systematic image simulations, depicted in Figure S4, were conducted to achieve the best agreement with the experimental data. A Gaussian distribution was convolved into the simulations to incorporate the effective source size and probe instability. The full width at half maximum of the Gaussian distribution was determined so that the experimental and simulated ADF image intensity profile of the Ga/Ge column, indicated by the blue line in Figure S3b, matched. The full width at half maximum was set to 0.60 Å for layer 3 and 0.50 Å for layer 4.

To simulate the thermal diffuse scattering from atomic vibrations, we employed a frozen phonon model assuming an Einstein model with the atomic displacement parameters. We used 1000 configurations per probe position to ensure accuracy in the simulations. Crystallographic parameters and atomic displacement parameters for atoms other than Ba were obtained from the Rietveld analysis of synchrotron powder diffraction data at 300K[37]. For the Ba sites, isotropic and anisotropic atomic displacement parameters were used for the Ba1 and Ba2 sites, respectively, as noted in the main text. The Ba2 site is restricted to two degrees of freedom, considering only the diagonal terms, based on its symmetry and the results of the previous Rietveld analysis[37].

Section S3. Estimation of atomic displacement parameters for Ba1

Since no significant anisotropy was identified for the Ba1 site, for those sites we performed Bayesian estimation for the two annular regions of the segmented detector: layer 3,

4 in Figure S1d. The parameters for Bayesian estimation were site occupancy and isotropic atomic displacement parameter. Probabilities were calculated for a 0.08 Å square centred around Ba1 indicated by orange line in Figure S3.

Heat maps for a certain atomic column are shown in Figure S5a, b for the posterior probability of parameters calculated for different scattering angle regions in layers 3 and 4, respectively. Each of these results shows that the results for a single layer are linearly spread over the parameters with large probabilities, indicating that focusing on just one layer leads to ambiguous estimation. This suggests that it is difficult to determine whether the changes in the ADF intensity profile are due to occupancy or atomic displacement parameters. However, as shown in Figure S5c, by combining the probabilities computed from the two layers and calculating their simultaneous probabilities, it is possible to estimate the two parameters simultaneously.

Section S4. The effect of electron beam heating on atomic vibration

The effect of the incident electron beam on atomic vibrations can be discussed by estimating the temperature increase of the sample due to the electron beam. According to Fisher's calculation[44], the maximum temperature rise ΔT due to electron irradiation can be calculated according to the following equation[45]

$$\Delta T = \frac{I}{4\pi\kappa e}\frac{\Delta E}{d}(\gamma + 2\ln(b/a))$$

where I is the beam current, κ is the thermal conductivity, e is the electron charge, ΔE is the energy loss per electron in a sample of thickness d, γ is Euler's constant which equals 0.5772, b is the sample radius, a is the beam radius. Usually, the energy loss of an electron is negligible compared to its initial energy. Therefore, $\Delta E/d$ is assumed to be constant and is approximately equal to dE/dx, which is the stopping power for the electrons. The thermal conductivity of $Ba_8Ga_{16}Ge_{30}$ at room temperature is 2.1 W/mK[20]. In this experiment, the beam current is I = 9.8 pA, the sample radius is b = 5 μm and the beam radius is a = 100 pm. The stopping power of electrons can be calculated to be 1.4 eV/nm using the Bethe–Bloch equation, assuming the average excitation energy for electrons in the target is 353 eV in the Bloch approximation[45]. Based on the above calculations, the maximum temperature increase of the sample is 4.3 K. Therefore, the temperature increase due to the electron beam is estimated to be very small and should not affect the present observation of the anisotropy of atomic vibration and the measurement of the amplitude of atomic vibration.

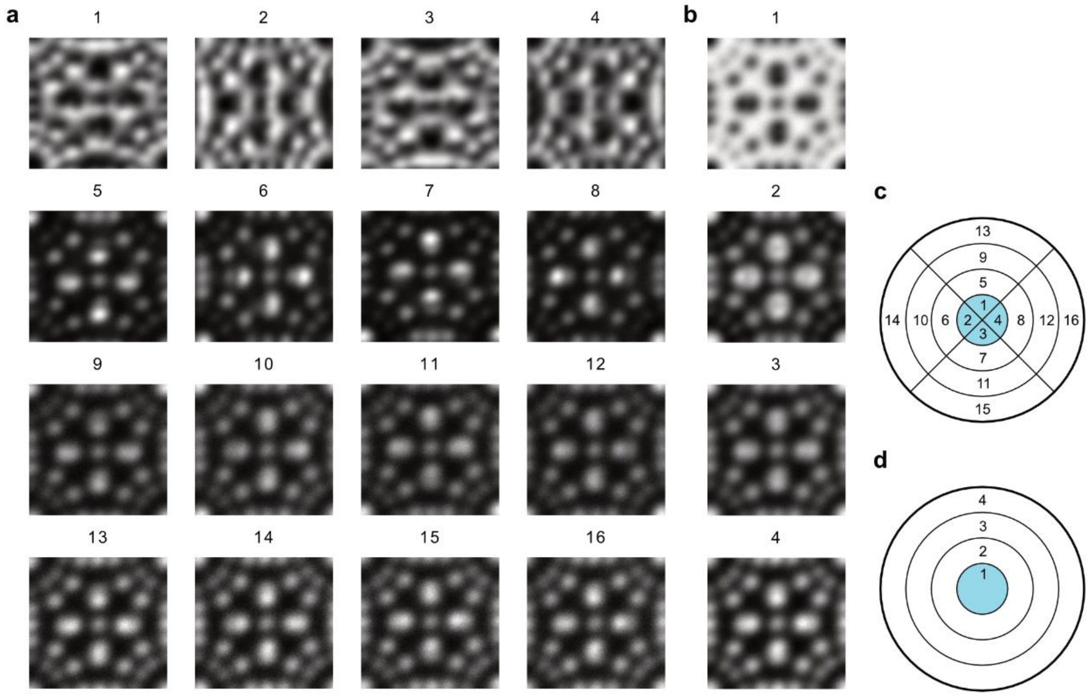

Figure S1. Repeat-unit averaged STEM image from each detector segment. a) STEM images from each detection segment. The number at the top of each image indicates the index of the segment in c. b) Layer images obtained by the addition of each detector segment image in the same annular region. The number at the top of each image indicates the index of layer in d. c) Segmented detector geometry showing the index of each segment. d) Segmented detector geometry showing the index of each layer. Blue circled area in c, d indicate a 20 mrad radius bright field disk.

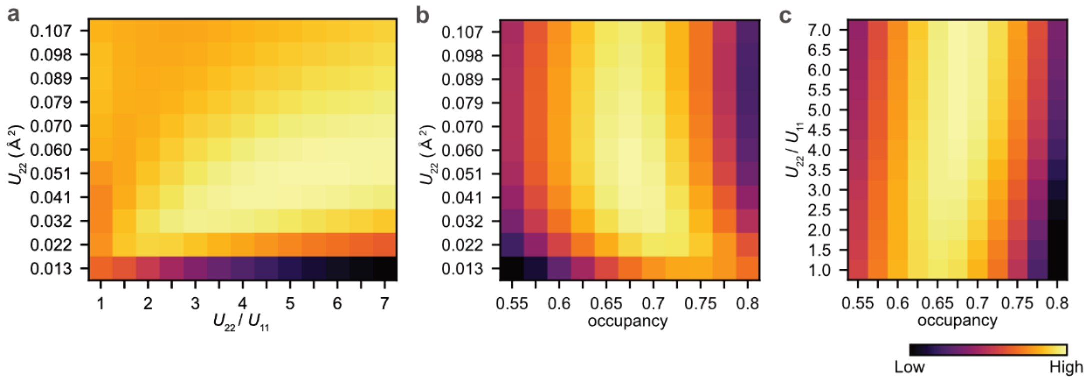

Figure S2. Heatmaps of the posterior probability of Bayesian estimation in Ba2 site. a-c) Heatmaps of the posterior probability cross sections for occupancy, U_{22}, and U_{22} /U_{11} intersecting at the point at which the probability is maximal. Brighter contrast indicates higher probability.

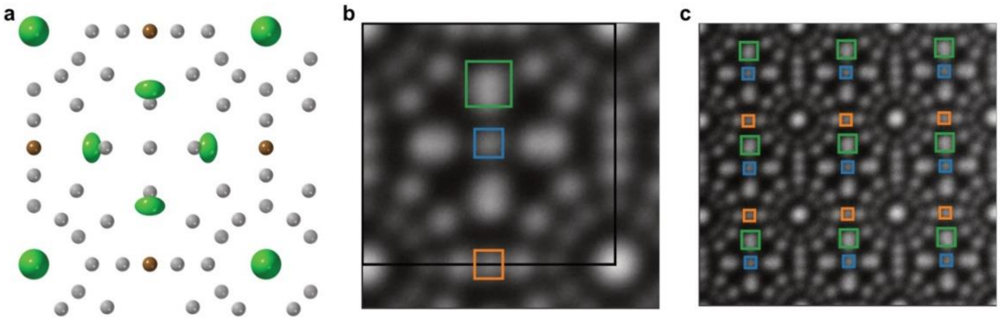

Figure S3. Areas of interest for the quantitative image analysis. a) Structural model of $Ba_8Ga_{16}Ge_{30}$ corresponding to the repeat-unit averaged ADF STEM image shown in b. b) The three regions of interest are indicated on the ADF STEM image. The orange square is 0.8 × 0.8 Å^2 centred on a Ba1 site. The green square is 2 × 2 Å^2 centred on a Ba2 site. The blue square is 0.8 × 0.8 Å^2 centred on a Ga/Ge column. The black square indicates the unit cell. c) Each region of interest is indicated on the non-averaged ADF STEM image.

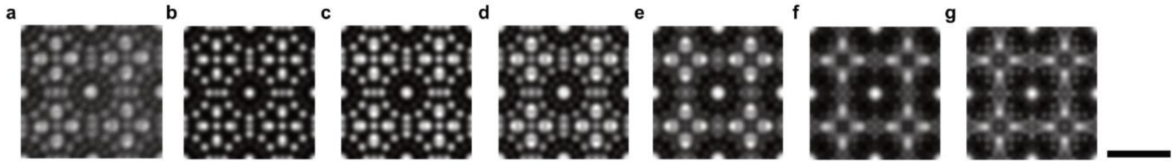

Figure S4. Low-angle ADF STEM images for sample thickness estimation. a) Experimental low-angle ADF STEM images at detection angles of 20-40 mrad. b-g, Simulated images at various sample thicknesses. Sample thickness is (b) 4 nm, (c) 6 nm, (d) 8 nm, (e) 10 nm, (f) 12 nm, (g) 14 nm. Other imaging conditions are consistent with the experiment. Scale bar at right is 1 nm.

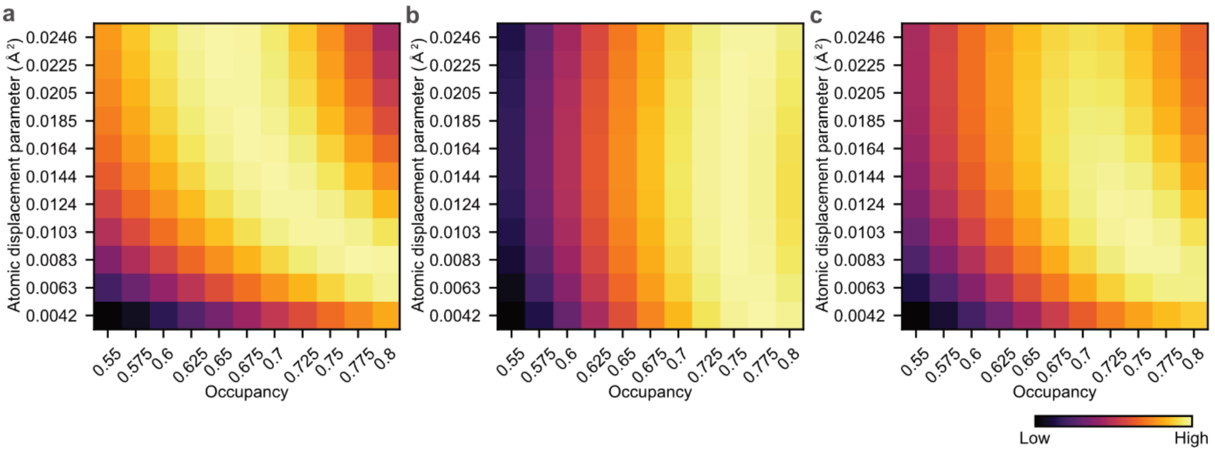

Figure S5. Heatmaps of the posterior probability of Bayesian estimation in Ba1 site. a-c) Heatmaps of the posterior probability of the two parameters for the Ba1 site: atomic displacement parameter and occupancy. Bayesian estimation was performed using the ADF image of (a) layer 3 or (b) layer 4. c) Simultaneous probability of the two probabilities computed in layers 3 and 4. Brighter contrast indicates higher probability.
